# Analysis of factors alleviating the psychological anxiety of transfer students in postgraduate re-exam based on structural equation model

**DOI:** 10.3389/fpsyg.2026.1862567

**Published:** 2026-09-09

**Authors:** Weiwei Yin, Fang Gao, Haishun Wang, Guowei Zhang

**Affiliations:** 1 School of Education, Anyang Normal University, Anyang, Henan, China; 2 School of Computer Science and Technology, Zhejiang University of Water Resources and Electric Power, Hangzhou, China

**Keywords:** mediating effect, postgraduate re-examination, psychological anxiety, self-efficacy, structural equation model, transfer students

## Abstract

**Background:**

Intense competition in China's postgraduate admission has resulted in a large number of students participating in the re-examination transfer process. High uncertainty, time pressure, and information asymmetry during transfer are linked to severe psychological anxiety that impairs students' mental health and academic adaptation. Empirical research on the anxiety mechanism and intervention paths for this group remains limited.

**Methods:**

Based on social cognitive theory (SCT), this study constructed a structural equation model (SEM) involving personal factors, external support, self-efficacy, and psychological anxiety. A questionnaire survey was conducted among transfer students from five universities in Henan Province, yielding 392 valid responses (including 187 male participants and 205 female participants), with an average age of 22.18 years old. Scales included the General Self-Efficacy Scale (GSES) and the 7-item Generalized Anxiety Disorder scale (GAD-7). Data were analyzed using SPSS and Amos for reliability, validity, SEM estimation, and Bootstrap mediation testing.

**Results:**

The measurement model showed good reliability and validity. The overall model fit was satisfactory (CMIN/DF = 1.958, RMSEA = 0.049, CFI = 0.948). All hypotheses were supported. Self-efficacy was negatively associated with anxiety (β = –0.431, *p* < 0.001). Personal factors and external support were positively associated with self-efficacy and negatively associated with anxiety. Self-efficacy significantly mediated the relationships between personal factors, external support, and anxiety.

**Conclusions:**

Personal cognition, proactive behavior, and support from schools, teachers, and families are negatively correlated with anxiety. Self-efficacy acts as a key mediator. These findings provide empirical evidence for targeted psychological interventions and support services for postgraduate transfer students.

## 1 Introduction

The entrance examination for graduate school is a widely-discussed topic in China's higher education (Tian and Gong, 2023). Though national authorities plan to expand postgraduate enrollment scales in the future (Liu, 2023), competition remains intense given limited annual admission quotas. Candidates who pass the national preliminary score line yet fail to secure re-examination spots at their target institutions may apply for vacant programs at other universities; this group of applicants is defined as *transfer students* (Liu and Jiang, 2006; Tian, 2020; Wang and Wang, 2008). Official 2023 enrollment data further demonstrates this competitive landscape: 1.6 million test-takers reached the national cutoff, while merely 1.1484 million master's places were available, leaving roughly 450,000 qualified applicants without initial admission opportunities (Wu, 2024).

The concentrated March–April transfer window is plagued by severe information asymmetry, tight timelines and unpredictable vacancies (Chen et al., 2024; Tan, 2020). Coupled with widespread graduate employment pressure, these uncertain contextual conditions are associated with a sharp rise in psychological anxiety among transfer applicants (Nie and Cheng, 2024; Yang, 2023). Within this narrow timeframe, students are forced to make high-stakes decisions that shape their long-term academic trajectories, which continuously amplifies their stress levels (Wei, 2022).

Existing literature identifies two core categories of protective resources that mitigate transfer-related anxiety. The first covers students' personal cognitive and proactive behaviors, including active information collection, rational goal formulation and positive self-regulation to lower uncertainty-induced distress (Zhu et al., 2010; Shen, 2006). The second encompasses multi-layered external support systems. University administrators deliver standardized, transparent transfer guidance and allocate adequate support resources to reduce applicant worries (Che et al., 2020); faculty provide integrated academic tutoring and emotional consolation for candidates (Wang, 2019); meanwhile, family members offer continuous understanding and encouragement throughout the adjustment phase (Fu, 2024; Zhou, 2025).

Beyond personal and environmental support, general self-efficacy acts as a critical psychological regulator of transfer students' anxious emotions (Zhang et al., 2024). It is conceptualized as individuals' core belief in their capacity to overcome obstacles and fulfill preset goals (Bandura, 1997). Therefore, this study constructs a structural equation model (SEM) featuring four latent constructs: personal factors, multi-source external support, self-efficacy and transfer anxiety. By empirically examining direct and indirect associations between these variables, this research aims to pinpoint core anxiety-mitigating mechanisms, deliver actionable coping strategies for applicants, and provide evidence-based service frameworks for universities to foster transfer students' psychological resilience and academic prospects.

## 2 Theoretical model and research hypotheses

Self-efficacy refers to an individual's belief and capacity to achieve goals (Bandura, 1997). It reflects a person's confidence and anticipation in accomplishing tasks and overcoming obstacles. A stable correlational connection exists between self-efficacy and people's behaviors, cognition, and emotions (Lippke, 2020). In terms of behavior, individuals with high self-efficacy are more likely to take proactive steps to pursue their goals and demonstrate greater perseverance and industriousness (Bandura, 1977). Concerning cognition, self-efficacy presents correlational links with people's self-assessment of capabilities. If we think we can solve a problem, we are more inclined to discover a solution (Bandura, 1997; Bandura and Locke, 2003). Regarding emotions, those with high self-efficacy are generally more positive, optimistic, and self-assured. They have trust in their ability to handle challenges and difficulties, and tend to report fewer anxious and depressive feelings. On the other hand, individuals with low self-efficacy often show correlational tendencies toward helplessness, dejection, and anxiety (Bandura and Locke, 2003).

Moreover, self-efficacy maintains close correlational connections with mental health conditions (Behr et al., 2025). Students with high self-efficacy tend to display stronger adaptive and problem-solving abilities in learning (Basileo et al., 2024). In terms of mental health, low self-efficacy often accompanies depressive and anxious states (Etherton et al., 2022). In psychology, anxiety is described as an emotional reaction, often an emotional experience of worry, uneasiness, and fear in response to a real or potential threat (Tang and Kuang, 2009). Anxiety-related feelings may correspond to various internal and external triggers, such as the fear of the unknown, concerns about the future, physical illness, social pressure, etc. (Penninx et al., 2021). When people feel anxious, they may experience restlessness, nervousness, fear, irritability, and may also be accompanied by physiological responses like an increased heart rate, sweating, and shortness of breath (Galla et al., 2014; Hawe et al., 2019).

There is a close association between self-efficacy and anxiety (Tahmassian and Jalali Moghadam, 2011). Research has documented a robust correlational link between self-efficacy and anxiety levels (Etherton et al., 2022; Guan, 2014; Siddiqui, 2015). People with high self-efficacy tend to manage challenges and stress more effectively, and this pattern aligns with lower anxiety levels. They have confidence in their abilities and believe they can handle various situations, so they are less likely to worry and become distressed (Fürtjes et al., 2023). In contrast, those with low self-efficacy are more liable to feel anxious. They lack confidence in their abilities and coping mechanisms, and such worry often coincides with elevated anxiety when facing difficulties (Gao, 2019; Schwarzer et al., 1999). Based on the above, we put forward the following hypothesis for the transfer students during this period:

1. H1: Transfer students' self-efficacy has a negative association with their anxiety.

In this study, the personal factors of transfer students we study include two core components: individual cognitive abilities and proactive actions. Among them, proactive actions are specifically manifested in transfer students taking initiative behaviors such as actively looking for relevant information, setting practical goals, and using self-care methods to preserve their mental health; individual cognitive abilities show negative correlational tendencies with anxious feelings.

Agency has been regarded as an ability exclusive to humans, with consciousness, intentionality, and free will as its prerequisites. It refers to the capacity of a subject to make autonomous choices and take actions (Lowe, 2008). Multiple correlational links can be observed between personal cognition, agency and self-efficacy (Bandura, 1990; Jiang, 2023; Schwarzer and Aristi, 1997). Firstly, accumulated personal knowledge and task experience show positive correlations with self-perceived capability. When an individual has in-depth knowledge and experiences in a specific area or task, they tend to develop confidence in their capabilities within that area. Such self-confidence corresponds to stronger self-efficacy and higher motivation when faced with relevant tasks. Secondly, agency maintains positive correlational connections with self-efficacy. Agency involves an individual's willingness and ability to initiate actions, pursue goals, and overcome obstacles.

In the transfer context, comprehensive mastery of transfer procedures and strong agentic tendencies tend to correspond to higher self-efficacy levels, and this combination correlates with milder anxious experiences during this critical period. Based on this, we propose the following hypothesis:

1. H2: Transfer students' personal factors have a positive association with their self-efficacy.

Insufficient knowledge of a specific matter often correlates with worry and anxious states, a psychological tendency defined as ambiguity aversion (Zhang, 2023). Clear understanding of a phenomenon's nature, background, potential outcomes and coping strategies correlates with greater capacity to tackle challenges and fewer anxious feelings.

For the transfer students, thorough familiarity with the postgraduate examination transfer process tends to correspond to lower anxiety during this period. By getting to know the various aspects of the transfer process, such as its requirements, procedures, and potential outcomes, transfer students can form a clearer mental image of what is to come, which correlates with a stronger sense of control and less uncertainty-induced fear. Therefore, we assume that:

1. H3: Transfer students' personal factors have a negative association with their anxiety.

The correlational link between external positive factors and individual self-efficacy carries substantial theoretical weight (Bandura and Locke, 2003; Kang, 2017). External positive factors, including institutional guidance from school administrators, academic and emotional support from instructors, as well as understanding and encouragement from families, jointly present correlational connections with transfer students' psychological states through two correlated pathways.

First, such multi-layered supportive resources correlate positively with self-efficacy. The consistent encouragement provided by schools, teachers and families acts as effective positive reinforcement that correlates with stronger self-belief (Guan, 2014). Prior successful coping experiences brought by external support also show positive correlations with confidence in handling subsequent challenges (Zhang and Schwarzer, 1995). A cooperative, supportive surrounding built via multi-stakeholder assistance cultivates a strong sense of belonging and self-worth, which correlates with higher levels of self-perceived capability (Guan, 2014). Accordingly, we put forward the corresponding hypothesis:

1. H4: The positive external factors of the transfer students have a positive association with self-efficacy.

Second, the same set of multi-source external support shows a negative correlation with transfer anxiety. During the stressful postgraduate transfer window, standardized procedural guidance from administrators correlates with less confusion; academic tutoring and emotional consolation from teachers align with reduced worry (Gao, 2019); steady family backing correlates with calmer mindsets amid uncertainties (Perkins et al., 2007). The coexistence of these supportive conditions correlates with milder anxious feelings among applicants, which leads to the hypothesis as follow:

1. H5: The positive external factors of the transfer students have a negative association with their anxiety.

In numerous academic studies, self-efficacy has emerged as a critical mediating variable, offering valuable perspectives on its moderating impact on various psychological and behavioral outcomes. This concept, which stems from Bandura's social cognitive theory (SCT), refers to an individual's belief in their ability to successfully carry out a specific task or attain a desired goal (Bandura, 1977, 1997; Bandura and Locke, 2003). As a mediating factor, self-efficacy connects the antecedent variables, such as personal characteristics, social support, or previous experiences, with the consequent outcomes, like motivation, performance, or well-being. For example, in educational research, it has been found that self-efficacy mediates the relationship between teaching strategies and student achievement (Guan, 2014; Siddiqui, 2015). Likewise, in workplace contexts, self-efficacy serves as an intervening link connecting leadership styles to employee job satisfaction and productivity. Moreover, in the field of health psychology, self-efficacy has been widely investigated as a mediating variable in health promotion and disease prevention (Mendes et al., 2024).

In short, treating self-efficacy as an intervening construct enables thorough unpacking of the intricate correlational links across diverse personal and situational variables tied to individuals' psychological states. Based on the conclusions from previous literature, the following two mediating effect hypotheses are proposed.

1. H6: Self-efficacy plays a mediating role between the personal factors of transfer students and their anxiety.
2. H7: Self-efficacy plays a mediating role between positive external factors and anxiety of transfer students.

Bandura's social cognitive theory (SCT) provides a unified theoretical framework to interpret the reciprocal associations uncovered in this study within China's unique postgraduate transfer context. Rooted in the tripartite interaction of personal agency, environmental input and self-perceived efficacy, SCT logically underpins all seven research hypotheses proposed above.

Specifically, individual proactive cognition and behavior (personal factors) generate mastery experiences to elevate self-efficacy and directly lower anxiety; multi-layered institutional, teacher and family support (external factors) deliver verbal persuasion and vicarious experience to boost efficacy beliefs while independently buffering anxious emotions. Self-efficacy further acts as the central cognitive mediator linking both personal and external antecedents to transfer anxiety, which explains why sufficient personal preparation and environmental support cannot fully relieve distress without robust self-confidence.

**Figure 1:**
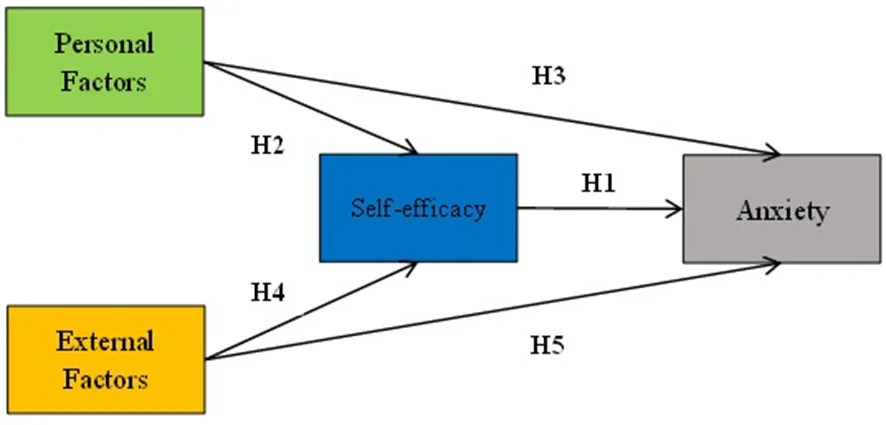
Conceptual theoretical model.

This research extends the conventional SCT framework to the high-uncertain, time-limited postgraduate adjustment scenario, supplementing localized empirical evidence for the theory's cross-cultural applicability. The conceptual model in Figure 1 visually systematizes these theoretically derived correlational pathways for intuitive interpretation.

## 3 Research design

### 3.1 Measurement instrument development

To ensure the reliability and validity of the four latent variables included in this study, we adopted a dual strategy for constructing measurement indicators: integrating well-established mature scales for two constructs, whose validity evidence has been fully verified in original literature (only sample-specific reliability indicators would be reported in subsequent analysis), and developing context-specific original items tailored to the scenario of Chinese postgraduate transfer for the remaining two constructs.

#### 3.1.1 Adopted standardized scales

Two internationally recognized, widely validated scales were directly employed to measure self-efficacy and psychological anxiety. The General Self-Efficacy Scale (GSES), originally developed by Schwarzer and colleagues in 1981, consists of 10 items and has been translated into 25 languages for cross-cultural research. This study adopted the Chinese revised version translated and validated by Wang and Liu (2000), whose stable psychometric properties among Chinese student populations have been repeatedly verified.

For anxiety measurement, the 7-item Generalized Anxiety Disorder scale (GAD-7) developed by Spitzer et al. (2006) was utilized. This self-reported scale assesses respondents' anxious symptoms including nervousness, worry, and restlessness over the past 2 weeks. Each item is scored on a 4-point Likert scale: 0 = Not at all, 1 = Several days, 2 = More than half the days, 3 = Nearly every day. The total score classification standards are as follows: 0–4 indicates no clinically significant anxiety; 5–9 mild anxiety; 10–14 moderate anxiety; scores above 15 represent severe anxiety.

#### 3.1.2 Development of customized personal factor and external factor scales

The two latent variables of Personal Factor and External Factor were self-developed strictly following standardized scale development procedures grounded in social cognitive theory and domestic research on postgraduate transfer adjustment. First, semi-structured interviews were conducted with transfer applicants, graduate admissions administrators and supervisors to extract practical descriptions and form an initial item pool. Second, an expert panel composed of educational psychology scholars and graduate management practitioners carried out content validity evaluation, revised ambiguous items and merged redundant items to obtain preliminary candidate items. Third, a pilot questionnaire containing tentative self-designed items and two mature scales was distributed to transfer students for item purification based on psychometric indicators. After screening, the retained formal measurement items of Personal Factor and External Factor were incorporated into the official questionnaire for formal data collection.

#### 3.1.3 Unified scoring standard

Except for the GAD-7 with its dedicated 0–3 scoring system, all remaining measurement items (PF, EF, GSES) adopted a unified five-point Likert scale ranging from 1 (strongly disagree) to 5 (strongly agree). This consistent scoring design accurately captures transfer students' real perceptions of adjustment pressure and supportive resources, providing solid data foundations for subsequent statistical analysis.

To systematically summarize the latent variables, item quantities and corresponding literature sources adopted in this study, Table 1 is presented below. The detailed items for each scale are provided in the Appendix.

**Table 1:** Variable construction and source.

Latent variable	Items	Source
Personal Factor	PF1-PF8	(Bandura, 1977; Du et al., 2022) and investigation summary
External Factor	EF1-EF6	(Bandura, 1977; Du et al., 2022) and investigation summary
Self-efficacy	SE1-SE10	Wang and Liu, 2000
Anxiety	AX1-AX7	Spitzer et al., 2006

### 3.2 Sampling strategy and participant demographics

#### 3.2.1 Sampling framework, inclusion and exclusion criteria

Participants were 2025 postgraduate re-examination transfer undergraduates recruited from five comprehensive undergraduate universities in Henan Province via convenience stratified sampling covering liberal arts, social science, engineering, and medical disciplines. Clear inclusion and exclusion standards were set to guarantee sample representativeness.

This study targeted fourth-year undergraduate participants who sat for the 2025 national postgraduate written examination, reached the corresponding national score line yet failed to secure re-examination opportunities at their originally chosen universities, actively gathered transfer-related information and intended to participate in adjustment interviews throughout March and April 2025, and were able to independently complete the full Chinese-language questionnaire. Exclusion criteria were set as follows: individuals admitted via postgraduate recommendation without taking the unified written test, those who had fully abandoned all transfer efforts prior to data collection, and international non-Chinese learners, so as to rule out anxiety confounds induced by language barriers. All questionnaires were designed and distributed in Mandarin Chinese, and all recruited participants were native Chinese speakers to guarantee consistent comprehension of survey items. Anonymous questionnaires were circulated via the WeChat Questionnaire Star mini-program across a 14-day data collection period, yielding a total of 410 raw responses.

#### 3.2.2 Data screening and missing data handling

Prior to carrying out formal statistical analysis, standardized data cleaning procedures were implemented with the support of SPSS 26 software. During the validity screening stage, a total of 18 questionnaires were discarded due to uniform repetitive Likert responses, overly short completion durations of less than 90 seconds, and inconsistent contradictory answers across survey items, which left 392 valid research samples and delivered a valid response rate of 95.61%. In terms of missing data handling, the result of Little's MCAR test verified that missing values were distributed completely randomly with a *p*-value of 0.214. Missing data only accounted for 1.17% of all total item responses, and the expectation-maximization imputation approach was utilized to supplement blank entries so as to avoid sample shrinkage caused by listwise deletion.

#### 3.2.3 Common method variance test

All data were collected via one-time self-reported questionnaires, which may lead to common method variance (CMV). Harman's unrotated single-factor test was performed on all items. The first extracted factor explained 36.7% of total variance, below the critical threshold of 40%, suggesting no severe common method bias in this study. Procedural anonymity and randomized item arrangement were also used to reduce consistent response biases in advance.

### 3.3 Ethical standards for questionnaire implementation

This research strictly followed standardized academic ethical norms and all survey procedures were approved by the Ethics Committee of the School of Education, Anyang Normal University prior to data collection. All questionnaires opened with a complete informed consent statement that clarified voluntary participation, full anonymity, and participants' unconditional right to withdraw at any time. No personally identifiable information (name, student ID, mobile number) was collected during data collection. All raw questionnaire data was stored on password-encrypted institutional servers to protect participant privacy. The entire survey design complied with academic moral codes, which strengthens the credibility of research conclusions (McDowell, 2006).

## 4 Research results and analysis

### 4.1 Reliability and validity test of the measurement model

For the two latent variables measured by self-developed scales, we first conducted a data suitability test for factor analysis via SPSS, targeting items about transfer students' self-cognition, proactive behaviors, and multi-stakeholder support from administrators, teachers and families. Two canonical tests were adopted: the KMO test and Bartlett sphericity test. The KMO value reached 0.922, which signifies excellent suitability for factor analysis. Meanwhile, the χ^2^ statistic of Bartlett sphericity test yielded a significance level of 0.000, satisfying the prerequisite for principal component analysis.

We further carried out principal component analysis combined with Varimax rotation on all items of the two self-designed scales to extract common factors, and the total explained variance is illustrated in Table 2. The two extracted principal components together explained 63.289% of total variance, covering most core information of the original observed variables.

**Table 2:** Total variance explained (self-developed scales).

Component	Extraction sums of squared loadings			Rotation sums of squared loadings		
	Total	% of variance	Cumulative %	Total	% of variance	Cumulative %
1	5.731	40.934	40.934	5.053	36.094	36.094
2	3.130	22.355	63.289	3.807	27.195	63.289

The rotated component matrix is displayed in Table 3. All measurement items had factor loadings above 0.70 on their corresponding latent dimension without obvious cross-loadings, which strongly supports the construct validity of the self-developed scales.

**Table 3:** Rotated component matrix (self-developed scales).

Item	Component 1	Item	Component 2
PF1	0.773	EF1	0.794
PF2	0.776	EF2	0.784
PF3	0.745	EF3	0.768
PF4	0.814	EF4	0.811
PF5	0.785	EF5	0.789
PF6	0.785	EF6	0.763
PF7	0.838		
PF8	0.784		

Extraction method: principal component analysis.

Rotation method: Varimax with Kaiser normalization.

Rotation converged in three iterations.

Specifically, the first extracted component is defined as Personal Factor, which reflects transfer students' individual characteristics, self-awareness, motivation and initiative in the postgraduate transfer process. The second component corresponds to External Factor, representing multi-layered supportive resources from school administrators, instructors and families that relieve transfer students' adjustment pressure.

### 4.2 Reliability and convergent validity of the overall measurement model

Subsequently, we proceed to the reliability evaluation of the proposed model. To ensure rigor and consistency with academic standards, we adopt the criterion that Cronbach's α coefficient should exceed 0.7, a threshold widely accepted in scholarly research. Our model comprises four key constructs: Personal Factors, External Factors, self-efficacy, and anxiety. From Table 4, we know that each of these constructs exhibits a Cronbach's α value exceeding 0.8, indicating a high degree of internal consistency and reliability.

**Table 4:** Reliability and convergence validity analysis of the measurement model.

Constructs	Item	STD.	**C.R**.	AVE	Cronbach's α
Personal factor	PF1	0.783***	0.917	0.580	0.915
	PF2	0.742***			
	PF3	0.719***			
	PF4	0.778***			
	PF5	0.760***			
	PF6	0.749***			
	PF7	0.821***			
	PF8	0.735***			
External factor	EF1	0.731***	0.881	0.552	0.878
	EF2	0.748***			
	EF3	0.737***			
	EF4	0.755***			
	EF5	0.760***			
	EF6	0.728***			
Self-efficacy	SE1	0.755***	0.946	0.635	0.945
	SE2	0.756***			
	SE3	0.801***			
	SE4	0.805***			
	SE5	0.807***			
	SE6	0.714***			
	SE7	0.872***			
	SE8	0.879***			
	SE9	0.780***			
	SE10	0.785***			
Anxiety	AX1	0.798***	0.900	0.563	0.899
	AX2	0.683***			
	AX3	0.728***			
	AX4	0.731***			
	AX5	0.772***			
	AX6	0.750***			
	AX7	0.784***			

Remark: *** indicates a significant correlation at the 0.001 level.

Finally, we proceed to test the convergent validity of the model. This step involves a Confirmatory Factor Analysis (CFA), which assesses the alignment between the theoretical constructs and the empirical data. It is crucial to establish the convergent validity of the measurement model as a prerequisite for further evaluation of the structural model. The findings, collated in Table 4, reveal that all constructs have standardized factor loadings surpassing the benchmark of 0.6 (denoted as STD. in Table 4), and these loadings are statistically significant. Furthermore, the Composite Reliability (C.R.) scores exceed 0.8, and the Average Variance Extracted (AVE) values are higher than 0.5.

These outcomes align with the established criteria outlined by Hair et al. (2010) and Fornell et al. (1996), which stipulate that: (1) factor loadings must be greater than 0.5, (2) Composite Reliability (C.R.) must exceed 0.6, and (3) Average Variance Extraction (AVE) values must be higher than 0.5. By fulfilling these criteria, our model demonstrates robust convergent validity, ensuring the accuracy and reliability of the subsequent structural model analysis.

### 4.3 Research based on structural equation model

#### 4.3.1 Overall fit indexes of structural equation models

**Figure 2:**
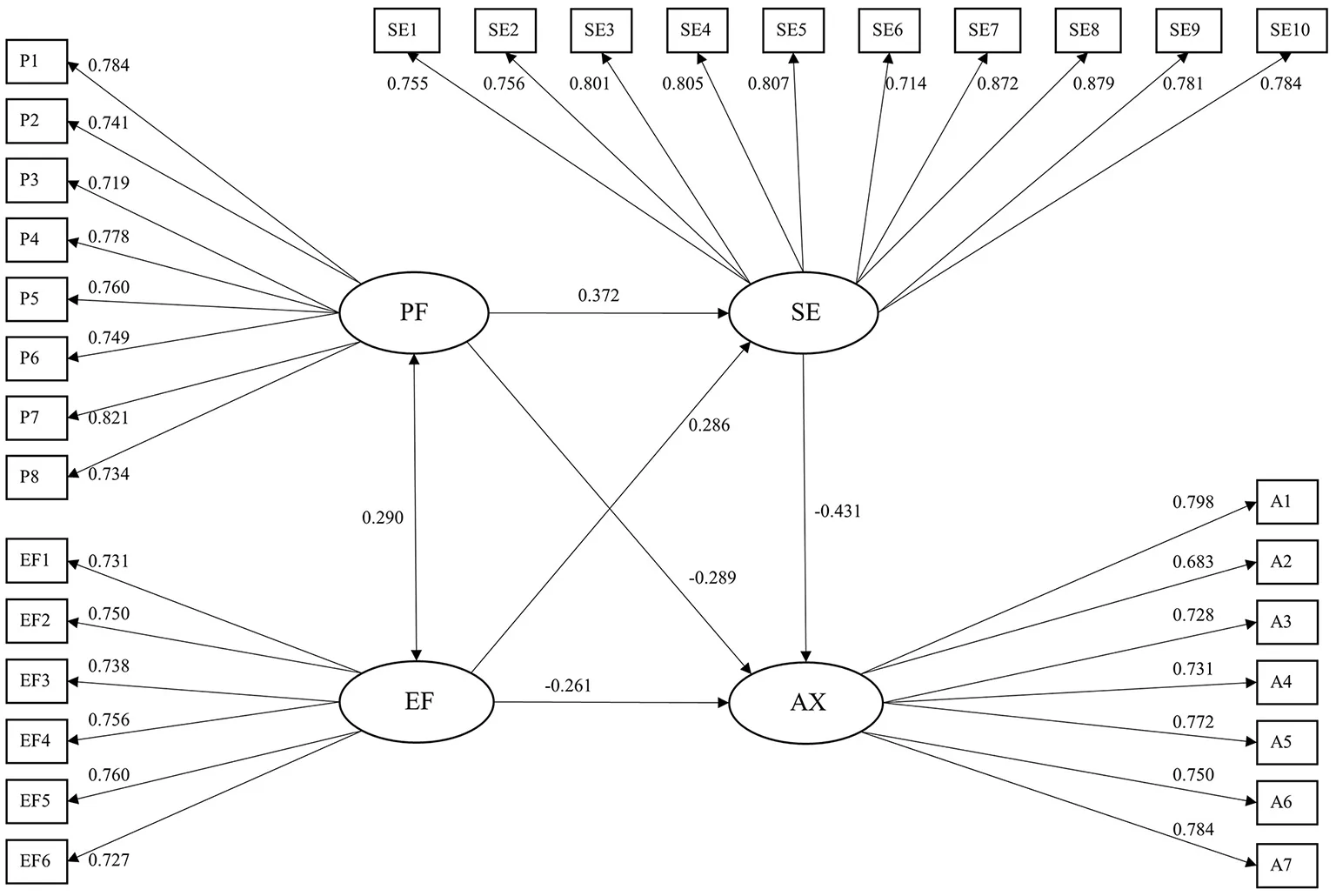
Structural equation model and path coefficients.

Utilizing the SPSS Amos software, we constructed a comprehensive structural equation model (SEM), as depicted in Figure 2. SEM was selected as the primary analytical method to achieve three core study goals: (1) simultaneously evaluate the reliability and convergent validity of four multi-item latent measurement constructs; (2) estimate simultaneous direct, indirect, and mediating correlational paths between exogenous, mediating, and endogenous latent variables within a unified statistical model; (3) conduct Bootstrap-based mediation testing to quantify indirect effect magnitudes, an analytical task that traditional regression alone cannot efficiently accomplish.

Prior to running model estimation in SPSS Amos, we comprehensively examined all core SEM statistical assumptions in a unified manner. The test for multivariate normality yielded a Mardia's coefficient of 21.47 with a critical ratio of 1.30 (below the 1.96 threshold), indicating no serious multivariate non-normal distribution. We further excluded multicollinearity risks, as correlation coefficients between all latent variables stayed under 0.70 and all variance inflation factors (VIF) were below 3.5. The anonymous cross-sectional questionnaire sampling design guaranteed independent observations and avoided biases from paired or clustered responses, while scatterplots of all paired observed items validated linear monotonic correlations among study.

The results of the structural equation model test, summarized in Table 5, provide detailed insights into the goodness-of-fit indices generated by Amos. When benchmarked against the established cut-off values outlined in the paper by Huang and Bentler (1999), most fit indexes fully meet the recommended thresholds. Notably, GFI = 0.884 and NFI = 0.899 slightly fall below the conventional 0.9 cutoff. GFI and NFI tend to produce downward biased estimates for models with numerous observed items; given the widely recognized standard of no less than 0.85 for social science structural equation models, these values are still acceptable. Meanwhile, all remaining absolute fit indices (*RMSEA* = 0.049, *SRMR* = 0.0504) and incremental fit indices (*CFI* = 0.948, *TLI* = 0.943) satisfy standard adequacy criteria which confirms that the constructed structural equation model adequately captures the correlational associations among the study's latent variables. This outcome validates the proposed theoretical framework and lays a rigorous statistical foundation for hypothesis testing, path coefficient interpretation, and mediation effect analysis in this work.

**Table 5:** Fit index of structural equation model.

Model fit indices	Cut-off value	Result
CMIN/DF	< 3	1.958
GFI	>0.9	0.884
AGFI	>0.8	0.866
RMSEA	< 0.08	0.049
SRMR	< 0.08	0.0504
CFI	>0.9	0.948
TLI	>0.9	0.943
NFI	>0.9	0.899
PNFI	>0.5	0.828
PCFI	>0.5	0.872

#### 4.3.2 Test results of research hypotheses

The relationships among latent variables, along with the estimated values of standardized path coefficients, CR (Critical Ratio) values, and the outcomes of hypothesis testing, are presented in Table 6. The CR value, a crucial indicator of parameter estimation in structural equation models, serves as a gauge to determine the significance of path coefficients in Amos. Specifically, the absolute value of CR exceeding 1.96 signifies a statistically significant path coefficient. The results in Table 6 show that all estimated path coefficients reach statistical significance at *p* < 0.001, reflecting stable correlational associations among the studied latent constructs. Here, PF represents Personal Factor, EF represents External Factor, SE represents Self-efficacy, and AX represents Anxiety. These variables and their interactions, captured through the path coefficients, form the backbone of the actual model depicted in Figure 2. The visualization of the model and the corresponding path coefficients provides a clear understanding of the complex relationships among the latent variables and how they contribute to the overall model.

**Table 6:** The test results of the hypotheses.

Hypothesis	Relationship	Standardized path coefficient	C. R. (Critical Ratio)	Result
H1	SE → AX	–0.431***	–8.177	Support
H2	PF → SE	0.372***	6.768	Support
H3	PF → AX	–0.289***	–5.973	Support
H4	EF → SE	0.286***	5.302	Support
H5	EF → AX	–0.261***	–5.517	Support

*** means significant at 0.001 level.

#### 4.3.3 Analysis of the mediating effect of self-efficacy

In this study, we employed the Bootstrap method within Amos to rigorously test the correlational mediation pathways, with 5,000 resamples and 95% bias-corrected and percentile confidence intervals adopted as standard analytical settings. This resampling technique delivers more precise estimates of indirect associations while accounting for sampling fluctuation and potential estimation bias, improving the statistical rigor of mediation tests. As summarized in Table 7, all bias-corrected 95% confidence intervals for indirect paths exclude zero, satisfying the core statistical threshold to confirm meaningful mediation patterns between exogenous latent constructs and transfer anxiety.

**Table 7:** Total indirect effects report.

Hypothesis	Mediation path	Effect	Effect value (standardized)	Bootstrap bias-corrected		Mediation effect testing
				Lower bounds	Upper bounds	
H6	PF → SE → AX	Total effect	–0.349	–0.229	–0.102	Support
		Direct effect	–0.289			
		Indirect effect	–0.160			
H7	EF → SE → AX	Total effect	–0.360	–0.187	–0.068	Support
		Direct effect	–0.247			
		Indirect effect	–0.113			

Beyond the significance test of indirect associations, we further quantified the proportion mediated to interpret the substantive magnitude of self-efficacy's intervening role. For the pathway linking personal factors to anxiety via self-efficacy (PF → SE → AX), the standardized indirect association equals –0.160 and the total correlational association reaches –0.349, yielding a mediated proportion of approximately 45.8%. This value reveals that nearly half of the overall association between personal proactive resources and transfer anxiety operates indirectly through self-efficacy. From the correlational perspective of social cognitive theory, active information seeking and thorough understanding of transfer rules show limited negative correlation with anxious feelings alone; these personal resources tend to link to milder worry via elevated self-perceived coping confidence, which demonstrates the correlational bridging role of self-efficacy between personal resources and transfer anxiety.

For the pathway from external support to anxiety through self-efficacy (EF → SE → AX), the standardized indirect association is –0.113 against a total correlational association of –0.360, corresponding to a mediated proportion of roughly 31.4%. Compared with personal resources, school, teacher and family support shows a weaker intervening link operating through self-efficacy. This distinction implies environmental support carries a stronger direct stress-buffering function independent of efficacy beliefs: institutional guidance and family comfort ease anxiety both by elevating students' self-confidence and by offering immediate tangible aid to resolve adjustment obstacles. Collectively, the divergent mediated proportions of the two pathways demonstrate that self-efficacy acts as a more dominant cognitive transmission channel for internal personal resources than for external environmental support, offering comprehensive theoretical insights into the triadic reciprocal mechanisms of social cognitive theory within China's postgraduate transfer context. The above quantitative and theoretical interpretations jointly reinforce the interpretive value of the mediation findings reported in Table 7.

## 5 Discussion

### 5.1 Causal interpretation constraints of cross-sectional data

Critical interpretive boundaries must be emphasized for all path coefficients reported in this cross-sectional survey design. While the SEM model tests theoretically hypothesized directional associations derived from social cognitive theory, cross-sectional snapshot data collected at a single time point cannot establish definitive causal or temporal ordering between variables. All standardized path values (PF → SE, EF → AX, etc.) only reflect concurrent correlational associations measured during the 2025 transfer window, not irreversible causal effects unfolding over time.

Three key limitations constrain causal inference in the present cross-sectional design. First, the absence of temporally separated measurements means personal factors, external support, self-efficacy and anxiety were not assessed sequentially across pre-transfer, mid-transfer and post-transfer stages, leaving us unable to verify whether strong personal agency precedes higher self-efficacy instead of reciprocal reverse associations—for instance, reduced anxiety might equally motivate students to actively collect transfer-related information. Second, several unmeasured confounding covariates, including undergraduate academic standing, household socioeconomic background and past examination setbacks, could simultaneously correlate with all four latent variables and artificially magnify the strength of the detected path coefficients. Third, the static one-time survey fails to capture dynamic fluctuations in anxiety, self-efficacy or perceived external support throughout the months-long transfer period, making it impossible to examine whether enhanced personal initiative or institutional support gradually alleviates anxiety over the course of the adjustment process.

All results below are interpreted as consistent with the theoretically proposed directional model, but not definitive proof of unidirectional causality. Longitudinal three-wave panel designs are required to rigorously validate the causal mediation pathways proposed in this paper.

### 5.2 Comparison with existing literature

#### 5.2.1 Self-efficacy and anxiety

Our finding that self-efficacy has a negative correlational association with anxiety (H1 supported) aligns with abundant prior research. For instance, studies in educational psychology have consistently shown that individuals with high self-efficacy, who believe in their ability to overcome challenges, experience less anxiety in academic pursuits like exams (Bandura, 1997; Wang and Liu, 2000). Consistent with cross-cultural nursing education research focused on academic assessment stress, Warshawski et al. (2019) conducted a cross-sectional survey of 240 Israeli undergraduate nursing students and empirically validated that elevated academic self-efficacy correlates significantly with reduced test anxiety, alongside supplementary evidence that social support further reinforces this protective psychological mechanism (Warshawski et al., 2019). This cross-cultural educational evidence echoes the core correlation uncovered among Chinese postgraduate transfer candidates: even though Western higher education systems do not operate a centralized postgraduate transfer matching scheme identical to China's, the inverse association between self-efficacy and academic assessment anxiety holds universally for students facing competitive, high-stakes evaluation outcomes. In the context of transfer students, this means those confident in navigating the transfer process feel less anxious.

#### 5.2.2 Personal factors and self-efficacy/anxiety

The positive correlational link between personal factors (encompassing knowledge of the transfer process and proactive agency) and self-efficacy (H2 supported) echoes theories on how personal cognition shapes self-belief. Just as students with a good grasp of course materials have higher self-efficacy in exams, transfer students who are well-informed about transfer procedures and take initiative boost their self-efficacy. Cross-cultural university research from Spain further corroborates this mechanism: students who adopt diversified proactive coping strategies including planning and information-seeking exhibit significantly higher general self-efficacy, verifying the universal positive link between personal proactive behaviors and self-efficacy across distinct academic contexts despite the absence of China's postgraduate transfer matching system in European higher education (Freire et al., 2020). Moreover, the negative correlational association between personal factors and anxiety (H3 supported) resonates with research on ambiguity aversion. When individuals understand a process (reducing ambiguity), their anxiety diminishes, which is consistent with findings that knowledge reduces fear in uncertain situations (FeldmanHall and Shenhav, 2019).

#### 5.2.3 Positive external factors and self-efficacy/anxiety

That positive external factors (teacher, school, and family support) show a positive correlational association with self-efficacy (H4 supported) is in line with social cognitive theory, which documents correlational links between social support and self-belief. Family encouragement, teacher guidance, and school resources all contribute to an individual's belief in their capabilities, similar to how workplace support enhances employee self-efficacy (Luthans et al., 2007). A recent United States mixed-methods doctoral study highlights the critical function of teacher-provided support: adequate teacher guidance and accommodation resources strengthen students' self-efficacy when coping with academic anxiety, even though the American higher-education system lacks China's postgraduate transfer mechanism (Ryals, 2025). The negative correlational link between positive external factors and anxiety (H5 supported) also aligns with social support and mental health scholarship, which documents how support systems buffer feelings of stress and anxiety (Cohen and Wills, 1985).

#### 5.2.4 Mediating role of self-efficacy

The confirmation that self-efficacy mediates the relationships between personal factors and anxiety (H6 supported), and positive external factors and anxiety (H7 supported) is consistent with the mediating function of self-efficacy identified across multiple educational and healthcare contexts. For example, in health behavior research, self-efficacy mediates the correlational link between hope and health-promoting behaviors (Chen et al., 2025). Cross-cultural evidence from Türkiye further reinforces the universal psychological pathway: a recent nursing student study verified that self-efficacy fully mediates the link between digital literacy and AI-related anxiety, demonstrating this mediation mechanism holds in foreign student samples even without China's unique postgraduate transfer policy (Akay et al., 2026). Here, self-efficacy serves as a correlational bridge connecting personal knowledge and external support to lower anxiety levels.

### 5.3 Practical implications

Theoretically, this study expands the applicable scope of social cognitive theory within the high-pressure context of China's postgraduate transfer, detailing the correlational interactive relationships among personal proactive resources, multi-layered external support and self-efficacy that correspond to transfer students' anxious states, thus enriching cross-cultural empirical research on academic adjustment-related psychological distress. Based on the correlational patterns observed in the present data, hierarchical practical suggestions covering universities, instructors and families are put forward.

Universities may build permanent real-time information platforms to release updated transfer quotas, re-examination resources and supervisor contact information, which correlates with less information asymmetry. Recordable transfer seminars and one-on-one consulting sessions can be scheduled regularly, as such activities correspond to higher levels of student initiative and self-perceived capability accumulated through practical adjustment experience. Discipline-specific transfer advisors are also recommended to deliver tailored assistance including document revision and school communication coordination. For mental health services, institutions could design customized support schemes for transfer applicants rather than generic exam counseling, such as group sessions focused on self-efficacy development and brief psychological intervention for participants with elevated GAD-7 scores. Regular full simulated re-examinations are also advisable, since simulated adjustment experience correlates with stronger self-belief and milder uncertainty-linked anxious feelings.

Instructors function as important sources of verbal affirmation; combining academic tutoring on review planning and institutional selection with sustained emotional encouragement while helping students reach out to supervisors correlates with greater applicant self-confidence and lower worry about adjustment progress. In addition, schools can cooperate with families by distributing targeted brochures explaining full transfer procedures and candidates' typical psychological tendencies, to guide caregivers to deliver understanding encouragement rather than overwhelming pressure. Consistent supportive input from families, schools and teachers forms a multi-dimensional support system that shows negative correlations with transfer-related anxiety.

The multi-stakeholder supportive framework outlined above aligns with patterns of milder anxious emotions and better psychological adaptation among transfer applicants in the correlational data of this research.

## 6 Conclusions and suggestions

This study constructs a structural equation model based on social cognitive theory to explore the anxiety mitigation mechanism among postgraduate transfer students, taking personal factors, external support, self-efficacy and psychological anxiety as core latent variables. The empirical results verify all seven proposed hypotheses. Personal factors and multi-layered external support can directly reduce transfer anxiety, and both exert significant indirect inhibitory effects on anxiety via the mediating role of self-efficacy; among them, self-efficacy serves as a more prominent cognitive transmission channel for individual proactive resources.

In general, transfer students with adequate self-perception, active coping behaviors and sufficient multi-stakeholder support tend to hold higher self-confidence and suffer milder anxiety symptoms. Enhancing students' self-efficacy is the core pathway to ease adjustment-related negative emotions. This research expands the application boundary of social cognitive theory in China's postgraduate admission adjustment scenario, and provides empirical evidence for multi-subject psychological intervention work targeting transfer applicants.

This study also has certain limitations. Restricted by cross-sectional survey design, we cannot draw definitive causal inferences among variables; the sample is only recruited from universities within a single province, which limits the generalizability of research findings; besides, measurement bias may exist in the self-developed scales. Future research can adopt longitudinal tracking design, expand sampling scope and combine mixed research methods to further optimize relevant theoretical models.

## Data Availability

The raw data supporting the conclusions of this article will be made available by the authors, without undue reservation.

## Appendix

The following lists the four scales used in this article.

## Personal factors

PF1. I am very familiar with the postgraduate re-examination transfer process.PF2. I know the key timelines for postgraduate re-examination transfer.PF3. I am very familiar with the basic eligibility requirements for postgraduate re-examination transfer.PF4. I clearly understand all available channels to obtain postgraduate transfer information.PF5. I have confirmed my target universities for postgraduate re-examination transfer.PF6. I have fully prepared all documents required for postgraduate re-examination transfer.PF7. I can actively seek out and collect abundant postgraduate transfer information.PF8. I take the initiative to contact universities and supervisors that accept transfer applicants.

## External factors

EF1. The teacher's analysis and guidance on my grades and the universities I applied for were very helpful to me.EF2. The re-examination transfer lectures and other activities carried out by the school are very helpful to my transfer.EF3. The subject review and guidance sessions carried out by the school are very helpful to my postgraduate transfer.EF4. The simulated re-examination conducted by the school is very helpful to my transfer.

EF5. My family members are very supportive of my postgraduate entrance examination transfer.EF6. Teachers help me contact the graduate school and supervisors, which is very helpful.

## Self-efficacy

SE1. I can always solve problems if I try my best.SE2. Even if others oppose me, I still have the means to get what I want.SE3. It's easy for me to stick to my ideals and achieve my goals.SE4. I am confident that I can deal effectively with any unexpected event.SE5. With my intelligence, I can handle the unexpected.SE6. If I put in the necessary effort, I'm sure I can solve most of the problems.SE7. I can face difficulties calmly because I trust in my ability to deal with problems.SE8. When faced with a difficult problem, I can usually find several solutions.SE9. When there is trouble, I can usually think of some way to cope.SE10. No matter what happens to me, I can handle it well.

## Anxiety

Over the last 2 weeks, how often have you been bothered by the following problems?AX1. Feeling nervous, anxious or on edge.AX2. Not being able to stop or control worrying.AX3. Worrying too much about different things.AX4. Trouble relaxing.AX5. Being so restless that it is hard to sit still.AX6. Becoming easily annoyed or irritable.AX7. Feeling afraid as if something awful might happen.
